# Learning reduced kinetic Monte Carlo models of complex chemistry from molecular dynamics

**DOI:** 10.1039/c7sc01052d

**Published:** 2017-06-19

**Authors:** Qian Yang, Carlos A. Sing-Long, Evan J. Reed

**Affiliations:** a Institute for Computational and Mathematical Engineering , Stanford University , Stanford , 94305 , USA . Email: qianyang@stanford.edu; b Mathematical and Computational Engineering , School of Engineering , Pontificia Universidad Catolica de Chile , Santiago , Chile . Email: casinglo@uc.cl; c Department of Materials Science and Engineering , Stanford University , Stanford , 94305 , USA . Email: evanreed@stanford.edu

## Abstract

We propose a novel statistical learning framework for automatically and efficiently building reduced kinetic Monte Carlo (KMC) models of large-scale elementary reaction networks from data generated by a single or few molecular dynamics simulations (MD). Existing approaches for identifying species and reactions from molecular dynamics typically use bond length and duration criteria, where bond duration is a fixed parameter motivated by an understanding of bond vibrational frequencies. In contrast, we show that for highly reactive systems, bond duration should be a model parameter that is chosen to maximize the predictive power of the resulting statistical model. We demonstrate our method on a high temperature, high pressure system of reacting liquid methane, and show that the learned KMC model is able to extrapolate more than an order of magnitude in time for key molecules. Additionally, our KMC model of elementary reactions enables us to isolate the most important set of reactions governing the behavior of key molecules found in the MD simulation. We develop a new data-driven algorithm to reduce the chemical reaction network which can be solved either as an integer program or efficiently using L1 regularization, and compare our results with simple count-based reduction. For our liquid methane system, we discover that rare reactions do not play a significant role in the system, and find that less than 7% of the approximately 2000 reactions observed from molecular dynamics are necessary to reproduce the molecular concentration over time of methane. The framework described in this work paves the way towards a genomic approach to studying complex chemical systems, where expensive MD simulation data can be reused to contribute to an increasingly large and accurate genome of elementary reactions and rates.

## Abstract 2

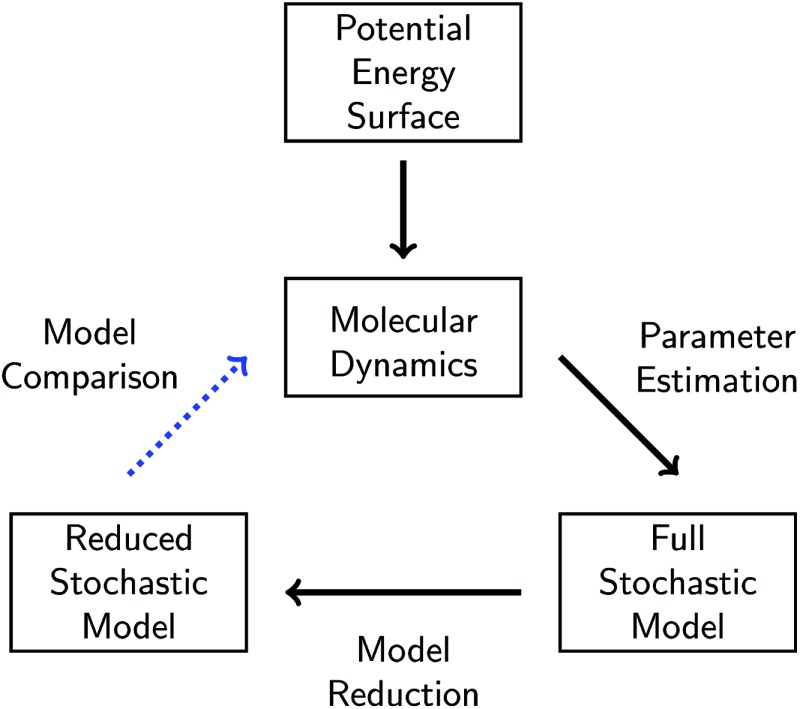
We propose a novel statistical learning framework for automatically and efficiently building reduced kinetic Monte Carlo (KMC) models of large-scale elementary reaction networks from data generated by a single or few molecular dynamics simulations (MD).

## 1 Introduction

In many fields of chemistry, biology, and materials science, the atomistic behavior of complex systems is studied using molecular dynamics (MD) simulations. These computations may involve thousands of atoms, and the resulting data is both detailed and complex, often corresponding to hundreds of molecular species undergoing thousands of reactions. Understanding the key chemical processes underlying this rich collection of data has thus become a grand scientific challenge.

On the other hand, MD simulations often require weeks of computation on high-performance parallel machines to produce data for a timeframe of merely nanoseconds or less. For many physical phenomena of interest, these time and system size restrictions present a significant limitation for meaningful modelling. Thus in recent years, there has been a growing need for scale-bridging models and algorithms that can enable faster and larger simulations without diminishing accuracy, and which exhibit useful predictive capabilities under different thermodynamic conditions.^1^

One important way to reduce atomistic simulations both computationally and as a physical model are kinetic Monte Carlo (KMC) methods.^2^ Given a set of states and transition rates between them, KMC methods enable stochastic simulation of a system's traversal through the given states over time. Because the simulations are now occurring on the timescale of transitions between states rather than atomic vibrations as in molecular dynamics, KMC methods greatly increase the speed of computation over the same timeframe. However, these methods require all of the states and transition rates to be known *a priori*; usually these are encapsulated by a small event table of key chemical processes derived from chemical intuition, with transition rates carefully computed from energy barriers derived using transition state theory, which can be very expensive. Such approaches quickly become intractable at high temperatures in condensed phases, where thousands of reactions or more can occur.

In this work, we propose a statistical framework for analyzing the complex chemistry simulated with molecular dynamics to build a KMC model corresponding to the same system. Rather than using a small event table of chemically intuited reactions with expensively calculated reaction rates, we use molecular dynamics data to generate a large event table of all observed elementary reactions with statistically estimated rates. We discuss in depth how bond duration should be chosen to optimize the predictive power of the KMC model, and demonstrate the ability of the resulting models to extrapolate in time. We then systematically reduce the model to gain chemical insight. Previously in Yang,^3^ an L1-regularization based model reduction algorithm was proposed. Here we describe how our data-driven approach first leads to an integer program, and how the L1-regularization based method described in Yang^3^ is a convex relaxation of the integer program. Our data-driven model reduction algorithm is computationally efficient, requires minimal *a priori* information about the chemical system, and employs a single parameter that controls the tradeoff between the size of the reduced KMC model and the resulting modelling error. We compare the predictive power of the integer programming and L1-regularization based methods for the first time with the simple reduction method of eliminating infrequent reactions. Our results are demonstrated throughout on a system of high temperature high pressure liquid methane, under conditions similar to shock compression. Liquid methane is thought to be a major component of gas giant planetary interiors, and thus understanding its chemistry has important applications to planetary physics.

Molecular dynamics simulations using *ab initio* potentials from electronic structure theory have recently been shown to reveal new reaction pathways in complex chemistry^4^ and to enable probing of high temperature high pressure conditions for which microscopic mechanisms are difficult to analyze experimentally.^5^ Researchers have also discovered ways to predict chemical reactions from reactants and reagents using neural networks.^6^ In combination with these techniques for finding new reactions, our statistical framework could eventually be used to build a comprehensive kinetic Monte Carlo model for complex chemistry that can be increasingly refined to include more elementary reactions and better rates, which can then be systematically reduced for particular systems of interest, enabling rapid simulation capabilities over a wide range of chemical compositions and thermodynamic conditions.

### 1.1 Background

In communities that study large-scale chemical reaction networks, model reduction is an important area of research. One common starting point is to model a system of interacting reactions as a deterministic set of ordinary differential equations. The combustion community, for example, has built sets of reference chemical reaction networks such as GRI-Mech 3.0 (ref. 7) consisting of hundreds of reactions with corresponding temperature and pressure-dependent reaction rates, which can then be simulated under different conditions using an ODE solver. The combustion community and others have studied model reduction from the viewpoint of solving an optimization problem over this set of coupled ODEs. Existing algorithms focus on various global optimization methods such as integer programming^8–11^ and genetic algorithms.^12^ While these methods have had some success, they are generally expensive and highly parameterized. Integer programming is NP hard, and therefore difficult to employ on arbitrarily large chemical reaction networks. Often, pre-reduction of the networks are necessary before optimization methods can be used efficiently. Genetic algorithms on the other hand often require extensive parameterization, which means the optimization problem must be fine-tuned for each individual system. This is a barrier to fast development of reduced models for systems for which chemical intuition is not readily available.

The biochemistry community has also studied the problem of model reduction. Some popular methods include lumping of species, graph theoretic approaches, and quasi-steady state approximations, as reviewed by Radulescu *et al.* ^13^ In contrast to combustion, where molecular species are generally studied in large molar quantities, many biochemical systems of interest involve small enough concentrations that the stochastic properties of the chemical system are important. Thus, in addition to modeling chemical networks as deterministic systems of ordinary differential equations, the biochemistry community also uses stochastic models, including both discrete-time Markov state models and continuous-time Markov models. When built directly from data, the finite state space for these models can be very large; thus both efficient parameter estimation and model reduction are very active areas of research.^14^

Finally, many scientific communities use sensitivity analysis to reduce event tables for kinetic Monte Carlo simulations.^15^ One commonly used method is Principal Components Analysis (PCA) of the rate sensitivity matrix,^16^ whose elements are the partial derivatives of the lognormalized species concentrations with respect to reaction rate parameters. Thresholds are chosen for the number of principal components to consider in the reduced mechanism, and also for identifying the significant elements of each principal component. This procedure must be done at multiple time points to build a collective reaction mechanism. The main drawback of this method is that it requires careful application of all of these different thresholds, and in fact some thresholds may need to be chosen simultaneously. It also requires many stochastic simulations to build each element of the sensitivity matrix at multiple time points, which can be prohibitively expensive for a large number of species and reactions. A modified version of this method in combination with some other approaches has been fully automated in Nagy and Turanyi.^17^ The required input is a full reaction mechanism.

### 1.2 Our approach

**Fig. 1:**
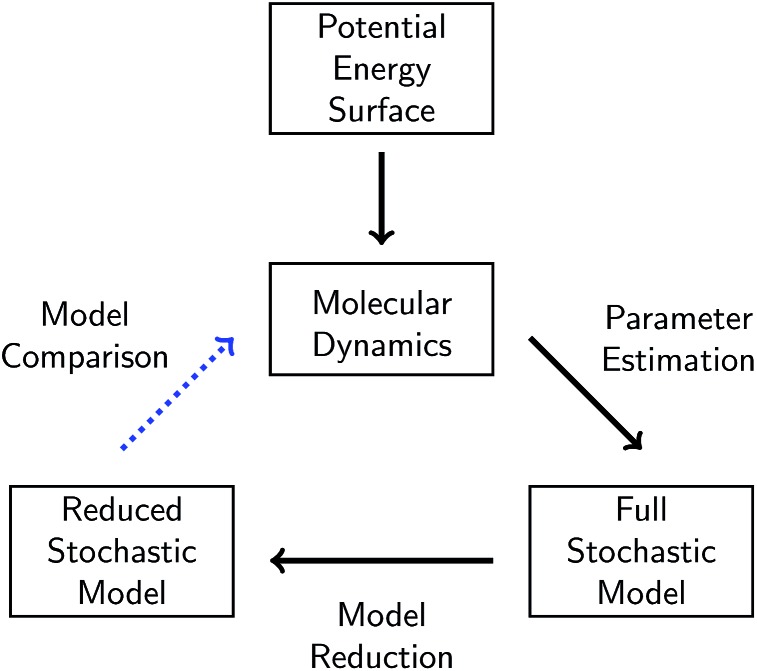
Schematic of our overall approach. A given chemical system is described by its potential energy surface. We first sample from this surface *via* molecular dynamics simulations. We then use parameter estimation to derive a stochastic model consisting of elementary reactions and corresponding reaction rates from the molecular dynamics data. This model is called the full stochastic model because it includes all reactions observed from the molecular dynamics simulation. We apply one of several model reduction techniques to reduce the full stochastic model by eliminating reactions. Finally, we compare the dynamics of the reduced stochastic model to the molecular concentration trajectories observed in the molecular dynamics simulations.

In this work, we present a systematic framework for building a reduced KMC model to represent any chemical system that can be modeled well by some chemical master equation. The full process is outlined in Fig. 1.

**Fig. 2:**
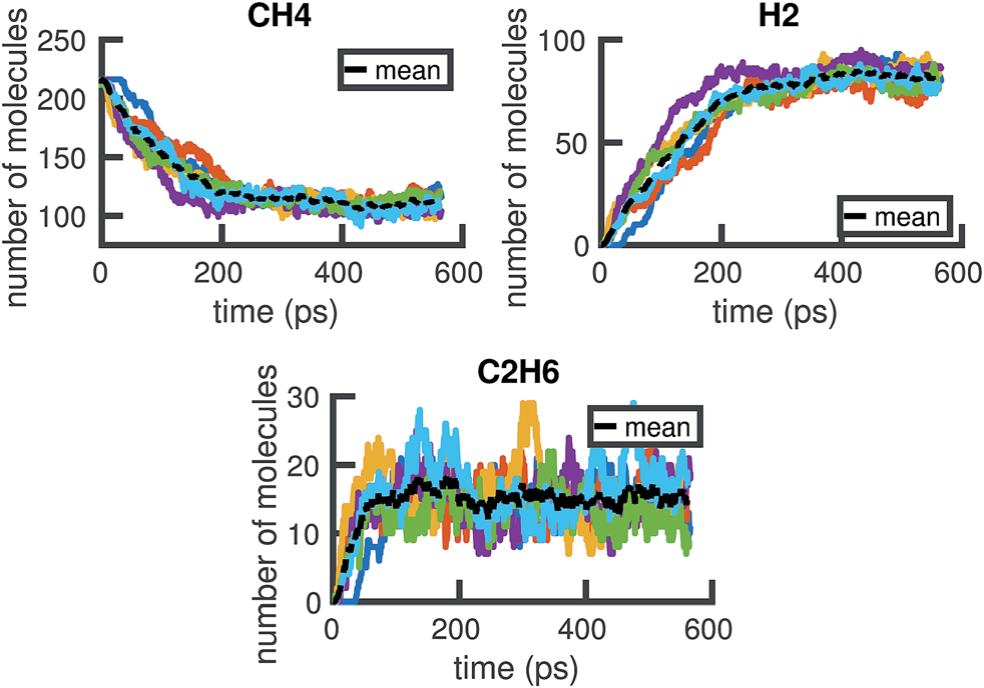
Six independent molecular dynamics simulations of the same system under the same thermodynamic conditions, resulting in somewhat different molecular concentration trajectories due to different initial velocities. Each colored line corresponds to a projected molecular concentration trajectory derived from a single molecular dynamics simulation. The dotted black line corresponds to the mean of these trajectories. We see that although the general trends are the same across simulations, the number of molecules at any given time can fluctuate between simulations. The average difference (in the root mean square sense) between each molecular concentration trajectory and the mean trajectory is about 8.0, 5.9, and 3.3 molecules for CH_4_, H_2_, and C_2_H_6_, respectively.

A given chemical system is characterized by its potential energy surface. We begin by sampling from this surface using molecular dynamics simulation, which produces a time series of the system as it traverses phase space. It is important to emphasize that this time series should be considered as only one sample of a trajectory on the potential energy surface. Molecular dynamics simulations are initialized with random initial velocities, and as such multiple runs of the same simulation can and do produce significant differences in the sampled trajectories, as shown in Fig. 2. In fact, the chaotic nature of molecular dynamics simulations ensures that computationally, no matter how small the differences in initial conditions, two distinct molecular dynamics simulations will diverge significantly from each other in phase space after a short amount of time.^18^ Note that these differences in phase space do not necessarily mean the macroscropic properties of the system have changed; this suggests there can be significant redundancies in representing a chemical system in phase space.

It may thus be informative to consider a transformation that collapses regions of phase space into points in molecular concentration space, separated by energy barriers. In this work, we first explore the process of building a chemical reaction network in molecular concentration space from a molecular dynamics simulation in phase space *via* parameter estimation. We define a chemical reaction network to be a set of elementary reactions and their corresponding rates of reaction. The use of elementary reactions ensures that the resulting network is applicable over a wide range of chemical compositions, whether or not the system is at equilibrium. To extract molecules and reactions from phase space data, we will use bond length and bond duration criteria. Then for a given set of reactions and molecular concentrations, we will use maximum likelihood estimation to estimate the corresponding rates of reaction.

The parameters of the chemical reaction network are both the set of elementary reactions, and their corresponding rates of reaction. The parameter estimation procedure thus involves all steps taken to determine both reactions and rates. We characterize the complexity of the reaction network by the number of parameters it has; thus the fewer the number of reactions, the less complex the model. We will show how bond duration in particular affects the number of reactions chosen.

The set of elementary reactions and reaction rates give rise to a full stochastic model of the chemical system where the probability of being in any given state in the space of molecular concentrations is governed by the chemical master equation.^19^ The chemical master equation can be simulated exactly using Gillespie stochastic simulation^20^ (equivalent to KMC), in a matter of minutes rather than weeks for the corresponding molecular dynamics simulation.

From the full stochastic model, we can then study how to reduce the chemical reaction network to only the set of elementary reactions that maximizes the predictive power of the model on the concentration trajectories of a particular set of significant molecules. When the set of significant molecules is considered to be all observed molecules, it enables reduction of noise from the system, *e.g.* it removes reactions in the derived chemical reaction network that may arise from atomic vibrations rather than actual elementary reactions. When the set of molecules is limited to a few key molecules, model reduction can isolate the portion of the reaction network most relevant to the dynamics of those molecules. When model reduction decreases the order of magnitude of the fastest rates of reaction in the system, it can also significantly speed up Gillespie simulation of the important dynamics. We will study the results of three different methods for model reduction: one naive method rooted in physical intuition, one systematic method based on statistics and optimization, and one computationally efficient method that well-approximates the systematic method.

In each step of this process, there will be error in the modeled system. First, the accuracy of the molecular dynamics simulations depends crucially on the accuracy of the potential function used to model the potential energy surface. Second, deriving the full stochastic model from the molecular dynamics simulation assumes, among other things, a well-stirred system. Finally, the reduced stochastic model is by construction a reduction most relevant for the region in concentration space under study, and may become less applicable the further away the trajectory of the system moves from the sampled region. These and other sources of error in each step of the approximation will be discussed in more detail below. Nevertheless, we will show that it is possible to derive a significantly reduced stochastic model that can approximately reproduce the molecular concentration trajectories of significant molecules derived from molecular dynamics, with the complexity of the system increasing as more molecular species are tracked.

## 2 Molecular dynamics simulation

The system we will use in this study is high temperature high pressure liquid methane, under conditions similar to that found in shock compression. We use LAMMPS^21,22^ to simulate a computational cell of 216 methane molecules at 3300 K and 40.53 GPa for approximately 0.55 nanoseconds. We use the ReaxFF potential with the parameters of Mattsson *et al.*,^23^ which has been shown to be able to capture complex chemistry under extreme conditions.^24^ The integration timestep is set to 0.12 femtoseconds. Six independent molecular dynamics simulations are generated under these same thermodynamic conditions by initializing each with different velocities. To check for system size effects, we also simulated a cell of 125 methane molecules under identical conditions, and found that the systems contained a comparable ratio of both small molecules such as CH_4_ and H_2_ and larger carbon clusters.

## 3 Full stochastic model

Given a single molecular dynamics simulation, we use bond length and duration criteria to compute the observed concentration of molecules and the observed set of reactions at every time step. From this information, we use maximum likelihood estimation to estimate the rate coefficients for each reaction. The set of observed reactions and corresponding rate coefficients define a chemical reaction network that can be simulated with the Gillespie Simulation Algorithm to satisfy the chemical master equation.

### 3.1 Bond length and duration criteria

**Fig. 3:**
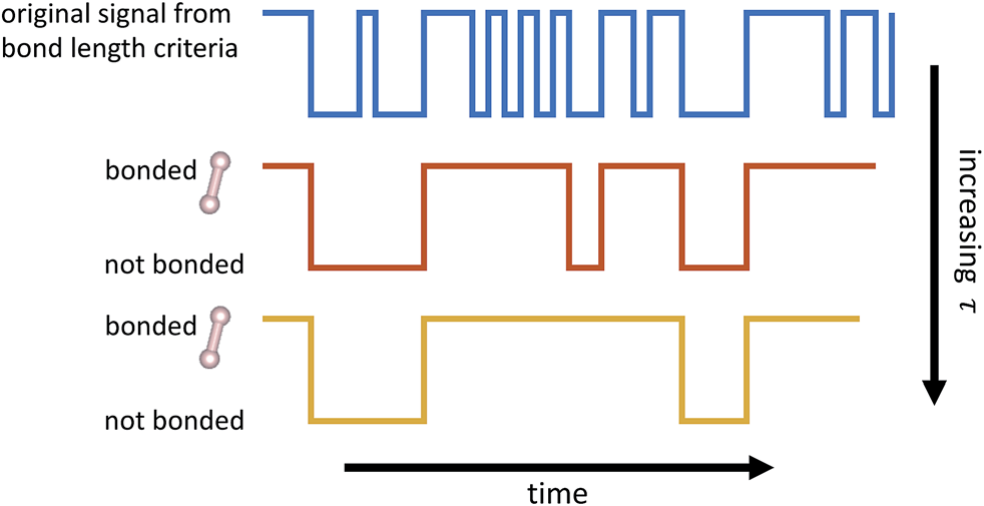
Schematic of how bond duration *τ* is used to smooth out the signal of whether or not atoms are bonded. Two atoms are not considered bonded unless the bond has endured for *τ* timesteps. Two atoms that were previously bonded are not considered unbonded unless the bond has been broken for *τ* timesteps. Note that as *τ* increases, fewer events are detected.

From the time series of atomic positions given by the molecular dynamics simulation, we identify molecular species and corresponding chemical reactions. Atoms are considered to be bonded if their distance is below a given threshold for a given duration of time, *τ*. Similarly, previously bonded atoms are not considered unbonded unless their distance is above a given threshold for a time period of *τ*. A schematic of this bond duration criteria is shown in Fig. 3. The bond length criteria we used is reported in previous work:^25^ 1.98 angstroms for C–C, 1.09 angstroms for H–H, and 1.57 angstroms for C–H. These values were obtained from radial distribution functions under similar thermodynamic conditions using the same potentials.

The bond duration criteria *τ* has an important effect on the chemical reaction network obtained. We note that it is important to choose *τ* such that the bond duration is short enough for all reactions to be considered elementary, but also long enough such that atomic vibrations that happen to extend past the given bond length are not included as reactions.^26^ Different bond duration criteria lead to different molecular concentrations, chemical reactions, and reaction rates. In particular, one naive way to reduce the number of distinct reactions observed, and thus the apparent complexity of the chemical system, is to increase *τ*; in the limit of infinite *τ*, no reactions will be observed.

**Fig. 4:**
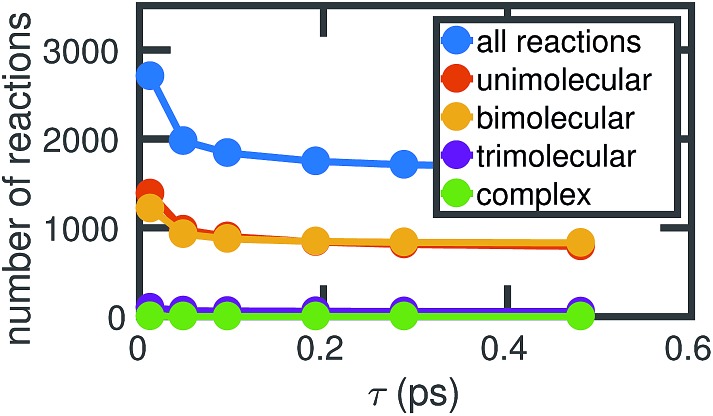
As the chosen bond duration *τ* increases, there are fewer of all types of reactions, thus decreasing the overall complexity of the system. For small *τ*, it is likely that many of the observed reactions actually correspond to atomic vibrations. Note that in our high pressure, high temperature system, some trimolecular and more complex reactions may reasonably be considered elementary.

Fig. 4 shows how the number of unimolecular, bimolecular, and other reactions observed in a single molecular dynamics simulation varies with *τ* (the counts are averaged over the six independent MD simulations computed). Note that there are a small number of trimolecular reactions, but only a nominal number of more complex reactions. Generally in the gas phase, elementary reactions are no more than bimolecular. In the high temperature high pressure liquid phase, it may be possible for trimolecular and higher order reactions to occur, but a large number of higher order elementary reactions is unlikely. Atomic vibrations are likely to account for many of the observed reactions at small *τ*.

**Fig. 5:**
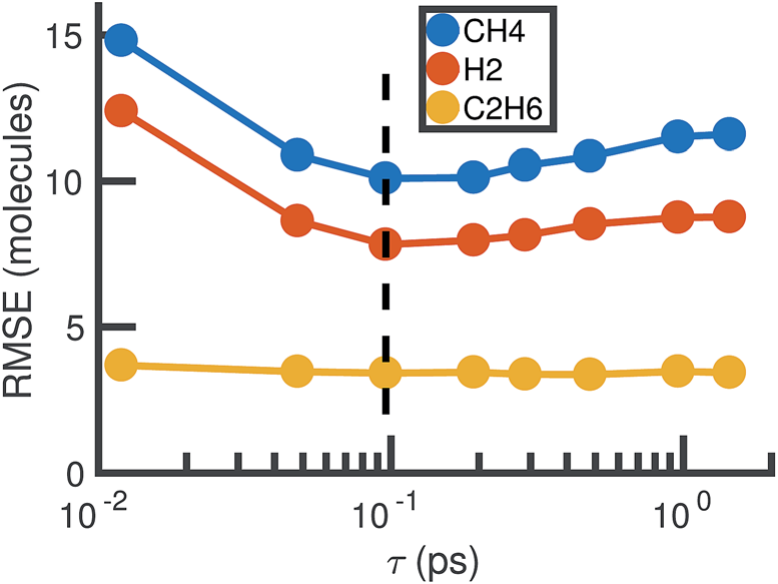
Root mean square error between molecular dynamics simulations and the corresponding stochastic model as a function of the bond duration criterion *τ* for the three highest concentration molecules. The error is computed according to eqn (6) and averaged over all six molecular dynamics simulations. For smaller *τ*, there is likely to be error from atomic vibrations. For larger *τ*, the reactions identified are unlikely to be elementary. The dotted line indicates the optimal choice of *τ* we use for the remainder of our study.

For each value of *τ*, we observe a set of reactions from the molecular dynamics trajectory and use maximum likelihood estimation (discussed below) to derive a corresponding set of reaction rates. This gives us a different stochastic model for each value of *τ*. Choosing the optimal value of *τ* is therefore a model selection problem: we select *τ* to maximize the agreement between the molecular dynamics simulation and the corresponding stochastic model. Fig. 5 shows how the choice of *τ* affects the error between the molecular concentrations computed from the molecular dynamics simulation and that simulated by the corresponding full stochastic model (we discuss in detail how error is computed below). The errors are averaged over the individual stochastic models corresponding to each of the 6 MD trajectories. Error for the three highest concentration stable molecules found in the system are shown. The effects of choosing too large (underfitting) or too small (overfitting) *τ* are most apparent for the two highest concentration molecules, CH_4_ and H_2_. In the remainder of this study, we will use a bond duration criteria of *τ* = 0.096 picoseconds, approximately corresponding to minimal error as shown in Fig. 5.

### 3.2 Chemical master equation

The stochastic model we will use is governed by the chemical master equation, which gives the probability at any time *t* of being in a given state in molecular concentration space.^19^

Consider a chemical system at thermal equilibrium and constrained to a constant volume. Suppose furthermore that the set of reactions that can occur in the system is not appreciably affected by the spatial position of the molecules; when this assumption is true we say that the system is *well-stirred*. Then we can associate with every reaction *j* a propensity function *a*_*j*_(**x**), which is defined such that *a*_*j*_(**X**(*t*))d*t* is the probability, given the vector of molecular concentrations **X**(*t*) at time *t*, that reaction *j* will occur once inside the fixed volume in the next infinitesimal time interval [*t*, *t* + d*t*). It can be shown^20^ that in a chemically homogeneous system, this propensity function is proportional to the number of possible combinations of the reactant molecules given the current concentrations of each molecular species, that is *a*_*j*_(**X**(*t*)) = *k*_*j*_ *h*_*j*_(**X**(*t*)), where *k*_*j*_ is the constant reaction rate coefficient and *h*_*j*_ is a function of the molecular concentrations that gives the combinatorial number of times that reactant molecules in the system could have achieved the given reaction. If reaction *j* is unimolecular, and there are *X*_*m*_(*t*) molecules of reactant molecule *m* in the fixed volume at time *t*, then *h*_*j*_(**X**(*t*)) = *X*_*m*_(*t*). If reaction *j* is a bimolecular reaction between two different species *m* and *m*′, then *h*_*j*_(**X**(*t*)) = *X*_*m*_(*t*)*X*_*m*′_(*t*), and if it is a bimolecular reaction between the same molecular species, 
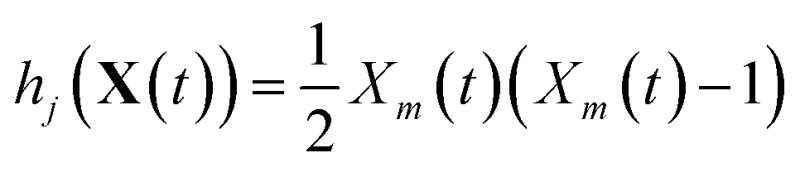
. In our datasets, we also observe a small percentage of trimolecular reactions. We treat these using the same combinatorial argument as above, *e.g.* for trimolecular reactions *m* + *m*′ + *m*′′ → products, we set *h*_*j*_(**X**(*t*)) = *X*_*m*_(*t*)*X*_*m*′_(*t*)*X*_*m*′′_(*t*), with suitable modifications if any of the reactants are the same molecular species.

An important observation to make about the propensity function is that while it is generally nonlinear in the molecular concentrations (except in the unimolecular reaction case), it is *always* linear in the reaction rate coefficients *k*_*j*_. This is a key property that we will exploit later for model reduction.

A system of reactions with corresponding propensity functions can be simulated *via* the Gillespie stochastic simulation algorithm described below to satisfy the chemical master equation exactly. In our framework, the system of reactions was selected *via* bond length and duration criteria. Therefore, it remains to derive propensity functions by estimating the reaction rate coefficients.

#### 3.2.1 Tau-leaping approximation

The propensity functions *a*_*j*_(**X**(*t*)) are defined with respect to infinitesimal time intervals [*t*, *t* + d*t*). However, for a molecular dynamics simulation with integration timestep Δ*t*, we only have information about the number of times each reaction occurs within time intervals of a fixed length Δ*t*. Thus, we cannot estimate the parameter *k*_*j*_ directly. Nevertheless, if we assume Δ*t* is small enough such that *a*_*j*_(**X**(*t*)) is approximately constant throughout [*t*, *t* + Δ*t*) for all reactions, then we can approximate the number of times each reaction *j* occurs in the time interval by conditionally independent Poisson random variables 𝒫_*j*_ with parameter *μ*_*j*_ equal to the propensity function times the time interval^27^

1 *μ*_*j*_(**X**(*t*)) = *a*_*j*_(**X**(*t*))Δ*t* = *k*_*j*_*h*_*j*_(**X**(*t*))Δ*t*

2
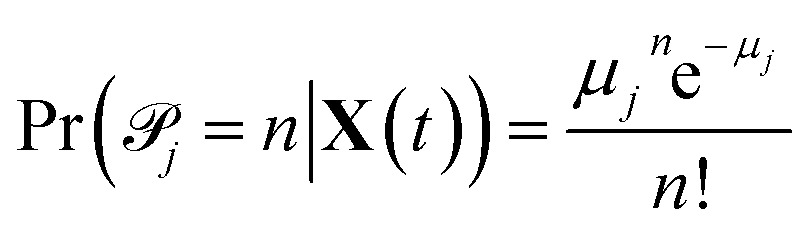



For each reaction *j*, we have an observation of 𝒫_*j*_|**X**(*t*) at all *t* for which *h*_*j*_(**X**(*t*)) ≠ 0. It is important to note that the 𝒫_*j*_ are all conditionally independent at any particular *t*; we assume that within the time interval [*t*, *t* + Δ*t*), the probability of any reaction *j* firing does not depend on whether or not any of the other reactions are also firing, but rather only on the molecular concentrations at time *t*. This approximation is true when Δ*t* is small enough so that few reactions are firing and the concentrations of all reactants is large enough that the few which are firing do not interfere with each other.

For some reactions, the number of observations is on the order of the total number of timesteps sampled in our molecular dynamics simulation. We can use any statistical point estimation technique to estimate *k*_*j*_ from these samples. For others, there is as few as just one observation of the random variable. In this case no point estimation technique will be reliable, but we will proceed with a likely overestimate of *k*_*j*_.

#### 3.2.2 Maximum likelihood estimation

We will use maximum likelihood estimation^28,29^ to estimate the *k*_*j*_. For each reaction *j*, our observations *n*_*j*_(*t*,*t*+Δ*t*) of 𝒫_*j*_|**X**(*t*) are conditionally independent but not identically distributed Poisson random variables. The likelihood of observing a particular sequence of *n*_*j*_(*t*,*t*+Δ*t*) can be expressed equivalently as3
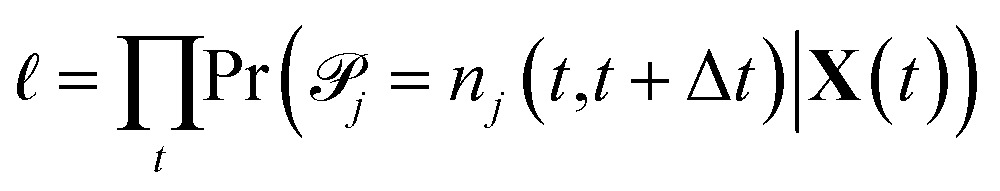
so that the log likelihood is given by4
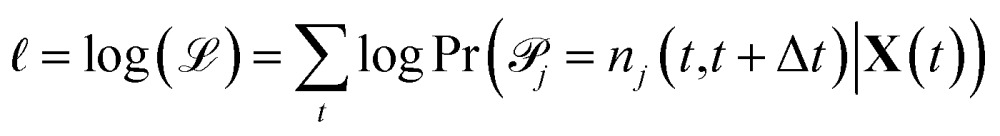



Maximizing 𝓁 and plugging in expressions 1 and 2 above, the resulting maximum likelihood estimate for the reaction rate coefficient *k*_*j*_ is5
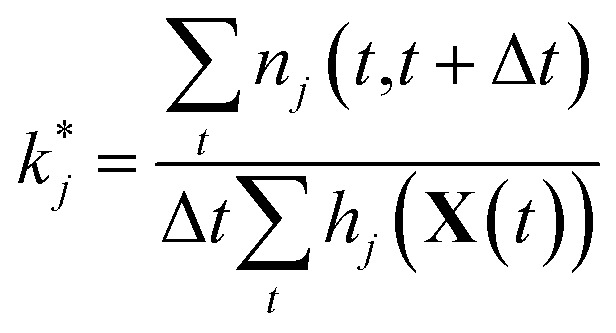



Since we are estimating each reaction rate separately, they will be estimated with different accuracy. In particular, we are likely to overestimate the rates of reaction for rarely possible reactions, since we will have very few nontrivial observations of 𝒫_*j*_|**X**(*t*) over which we are taking the maximum likelihood. As we will discuss below, model reduction helps to maximize predictive power by removing unimportant reactions for which we may have very noisy estimates of the reaction rate, increasing overall confidence in the model.

#### 3.2.3 Gillespie stochastic simulation

Given a set of reactions and reaction rates, we can simulate the chemical master equation exactly using the Gillespie algorithm,^20,30^ which models the time evolution of the chemical system in molecular concentration space by using random sampling to choose reaction events that cause transitions of the system from one concentration state **x** to another. The algorithm draws from the joint probability distribution *p*(*τ*,*j*|**X**,*t*), which is defined so that *p*(*τ*,*j*|**X**,*t*)d*τ* is the probability, given the current vector of molecular concentrations **X**(*t*) = **x**, that the next reaction to occur in the system will be reaction *j*, and will happen in the infinitesimal time interval [*t* + *τ*, *t* + *τ* + d*τ*]. At each step in the Gillespie simulation, we first choose the next reaction to occur based on the probability distribution
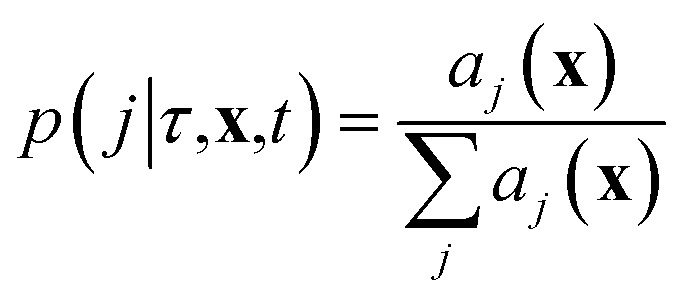
where we recall *a*_*j*_(**x**) are the propensity functions associated with each reaction. Then we compute the time until the next reaction occurs, which can be shown to follow the distribution of an exponential random variable*p*(*τ*|**x**,*t*) = *a*_0_(**x**)exp(–*a*_0_(**x**)*τ*)where 
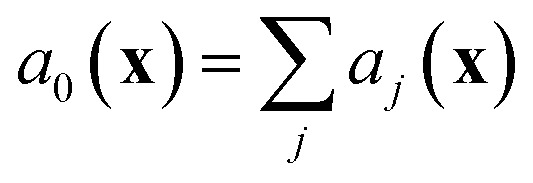
. We note that since any reaction that occurs over the course of the Gillespie simulation is by construction chosen from among the predetermined set of reactions and rates, this algorithm will not exhibit any events that were not observed in the molecular dynamics simulation.

### 3.3 Error metric

When comparing the molecular dynamics simulations and Gillespie stochastic simulations, we consider the time series of molecular concentrations for each molecule separately. We use the root mean square error between the molecular dynamics simulation and the mean of *S* stochastic simulations:6
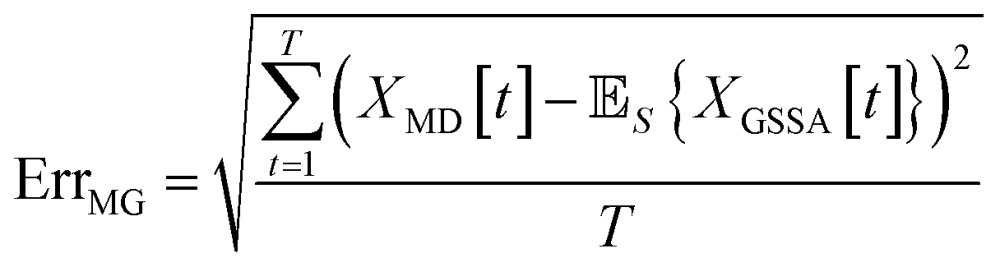



Here *T* is the total number of sampled timesteps of the molecular dynamics simulation; since the Gillespie simulation is technically a continuous time model, we can simply sample it at all times for which we have a corresponding MD sample. This error metric captures the difference between the full stochastic model and the molecular dynamics simulation of the potential energy surface, as well as stochastic fluctuations in a single MD simulation and in the finite set of *S* number of Gillespie simulations. We note that there may be some cancellation of error between these effects. We attempt to smooth out the stochastic fluctuations in the Gillespie simulations by averaging over *S* simulations, but in contrast there will be unavoidable fluctuations of the system captured by the single MD simulation. Note that the magnitude of this error term is dependent on the number of stochastic simulations *S* used to compute the mean stochastic trajectory, as well as the magnitude of fluctuations in the single molecular dynamics trajectory.

### 3.4 Results

**Fig. 6:**
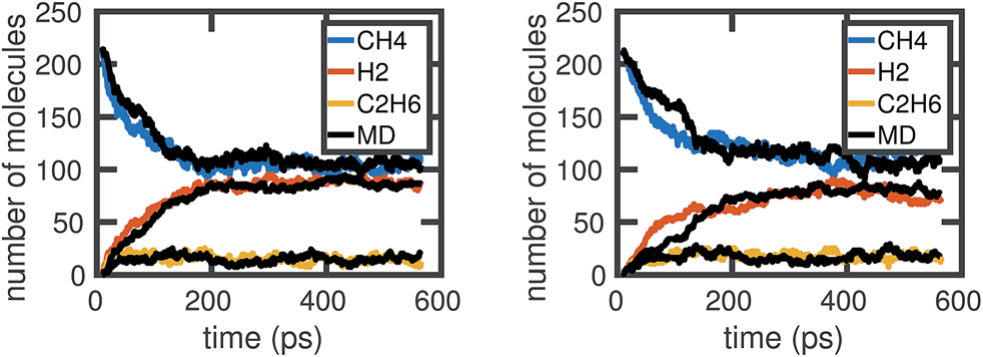
Two examples of Gillespie stochastic simulations of the chemical system compared to the molecular dynamics simulations they were trained from, when all of the molecular dynamics data are used. We see that both achieve reasonable agreement, especially in comparison to fluctuations between MD simulations as shown in Fig. 2. Differences in accuracy of the Gillespie simulations compared to the molecular dynamics are due to a variety of factors, including approximations made by the model as well as the stochasticity involved in a single simulation.

Fig. 6 shows two out of the six molecular dynamics simulations we computed, and corresponding Gillespie simulations of the stochastic model constructed from them. We can see that the stochastic model quite reasonably reproduces the molecular dynamics, albeit with differing levels of accuracy. This is both due to the stochasticity involved in a single simulation, as well as because the stochastic model includes several approximations, which we discuss below.

**Fig. 7:**
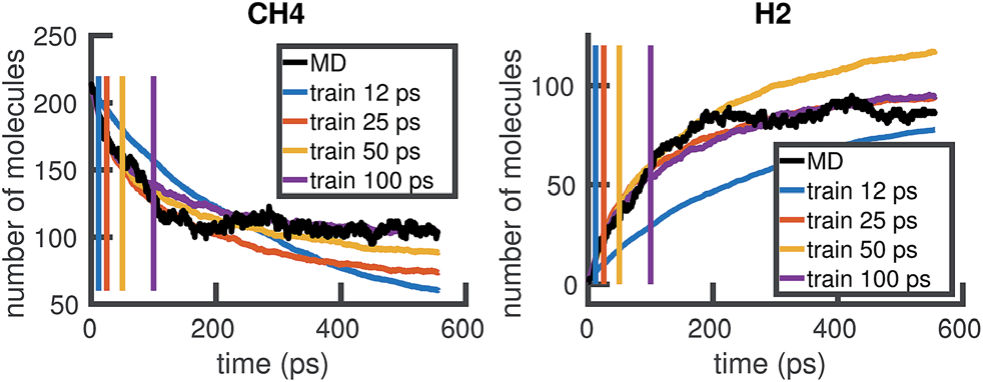
Time extrapolation: we use only the first 12, 25, 50, or 100 ps of available MD data to build a KMC model, then test model predictions at longer timescales. The colored lines show the molecular concentrations of CH_4_ (left panel) and H_2_ (right panel) averaged over 20 Gillespie simulations of the corresponding models. The black line corresponds to the true molecular concentrations from MD. While 12 ps of data is not enough to sufficiently model either molecule, 25 ps of data is enough to model CH_4_ for 200 ps and H_2_ for 500 ps.

**Fig. 8:**
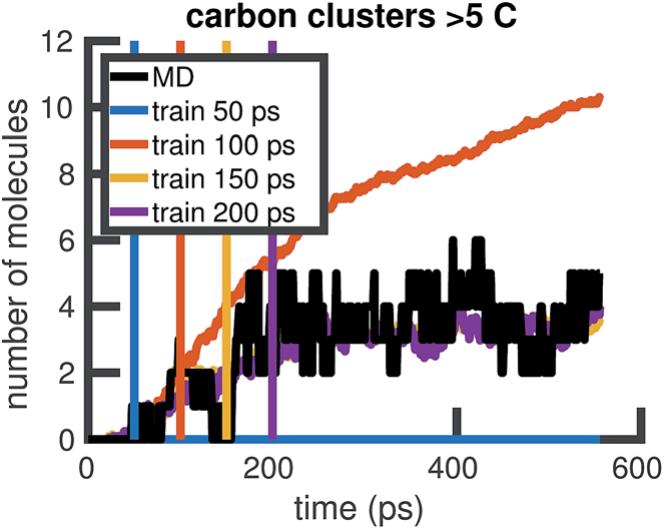
Time extrapolation of carbon-containing clusters: since larger molecules with more than 5 carbon atoms do not appear in the system until after about 50 ps, we find that training data from times out to 150 ps is needed to capture their presence and growth in the chemical system over 550 ps. All colored lines showing molecular concentrations of carbon clusters are averaged over 10 Gillespie simulations.

To understand whether the stochastic model is able to extrapolate in time and to different regions of molecular concentration space, we show in Fig. 7 the results of building the set of reactions and rates using only the first 12, 25, 50, and 100 picoseconds of molecular dynamics data. We see that with just the first 25 picoseconds of data, the model is able to predict the molecular concentrations of CH_4_ and H_2_ for up to approximately 200 picoseconds and 500 picoseconds, respectively. However, as Fig. 8 shows, data from times out to 150 picoseconds in the molecular dynamics simulation is needed to capture the growth of large carbon clusters, since reactions involving these clusters do not start appearing until later in time.

### 3.5 Limitations of the model

In constructing the stochastic model from molecular dynamics data, we have discussed above several approximations that were made. The most important was the tau-leaping approximation, which assumed the time interval [*t*, *t* + Δ*t*) is small enough and molecular concentrations are large enough such that all reactions occurring within the time interval are independent. We also assume the reactions are elementary so that the *k*_*j*_ are dependent only on the molecular concentration of the reactants. The estimated *k*_*j*_ are constructed with different confidence levels.

There are also several other approximations that come into play in the stochastic model. First, Poisson random variables can theoretically take on infinitely large positive integer values, albeit with very small probability. However, this is not the case with the number of reactions that are possible within a given time interval; that number is inherently limited by the number of reactant molecules available. Thus for example when there are only enough reactant molecules for one reaction to occur, we are actually observing a Bernoulli random variable. This may affect the accuracy of some of the *k*_*j*_, but the larger *μ*_*j*_(**X**(*t*)), the less this will have an effect.

Second, diffusion and local environment effects are simplified away from our model. Unlike the molecular dynamics simulation, the stochastic model has no knowledge of spatial arrangements of the molecules and assumes distance between reactants does not play an appreciable role in the rates of reaction.

## 4 Model reduction

Having built our full stochastic model, we now seek to reduce it by eliminating as many reactions as possible while minimizing any loss in the predictive power of the model. Why do we believe that this can be meaningfully achieved? Firstly, there is more information in the phase space data generated from molecular dynamics than there is in its coarse-grained projection onto molecular concentration space. The information that can not be transferred from our phase space data to our statistical model in molecular concentration space shows up as noise in the statistical model. Model reduction helps ensure that the model is not overfit to this noise. Furthermore, independently of modeling error, there are reactions that, for a particular region in concentration space, are physically unimportant to the overall concentrations of a subset of important molecules. We would like to find the smallest set of reactions that have predictive power for the dynamics of a subset of important molecules.

We explore three methods for model reduction. First, we use the naive method of simply eliminating infrequent reactions. Second, we set up model reduction as an optimization problem. The optimization problem can be solved exactly with integer programming, but any exact algorithm is NP-hard and quickly becomes computationally expensive as the size of the reaction network grows. We show how L1 regularization can be used to solve a convex relaxation of the problem that scales polynomially in the size of the network. The results from all three methods reveal that the majority of reactions can be eliminated from a stochastic model that seeks to predict the dynamics of only a few important molecules over a given time range, and that rare events do not play a large role in this chemical reaction network.

### 4.1 Count-based estimator

One intuitive way to reduce a given chemical reaction network is to simply remove the most infrequently observed reactions, until the dynamics of the system are affected beyond some error threshold. For some minimum count *f*, we eliminate reaction *j* from the stochastic model if7
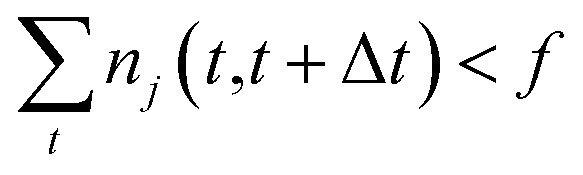
where *n*_*j*_(*t*,*t*+Δ*t*) are our observations of the number of times that reaction *j* occurred in the time interval [*t*, *t* + Δ*t*) as described above in Section 3.2.2. Note that this method is different from eliminating reactions with the lowest reaction rates *k*_*j*_, since *n*_*j*_(*t*,*t*+Δ*t*) depends on both the reaction rate *k*_*j*_ and the molecular concentration **X**(*t*) at time *t*.

In the results that follow, we take *n*_*j*_(*t*,*t*+Δ*t*) to be observations from the molecular dynamics simulations as described above. Note that Gillespie simulations also keep track of the reactions occurring at all times, so it is possible to alternatively observe *n*_*j*_(*t*,*t*+Δ*t*) from Gillespie simulations of the full stochastic model by binning the trajectory into fixed time intervals. This naive method is only possible when we have complete data for the number of each reaction occurring within every time interval. Since we are using molecular dynamics simulations or Gillespie stochastic simulations to generate our data, we can satisfy this requirement. This method would not be possible, for example, if our full system was described instead with a system of ordinary differential equations, or with incomplete experimental data.

It is interesting to consider how this method treats rare events. If rare but important events are observed in the given molecular dynamics trajectory, this method can only remove reactions that are more rare than the important rare event, thus possibly retaining a large number of frequent but unimportant reactions. However, if there are no important rare events observed in the system, then we can expect this naive estimator to perform quite well. Note that no model reduction technique based on sampled data can find rare events that were not observed. In fact, what we find is that the count-based estimator removes two types of reactions from the system: frequently possible but rarely observed reactions (reactions involving reactants with large concentrations but very low rates), and rarely possible but observed reactions (reactions involving reactants with very small concentrations). The former should have very good maximum likelihood estimates since the large concentrations of reactants means that we have many timesteps where *μ*_*j*_(**X**(*t*)) ≠ 0, and thus many samples of the random variable 𝒫_*j*_|**X**(*t*) = *n*_*j*_(*t*,*t*+Δ*t*). Conversely, the latter should have very poor, likely overestimated maximum likelihood estimates due to the small number of nontrivial samples of 𝒫_*j*_|**X**(*t*), and removing them helps increase the confidence in the overall dynamics of the remaining reaction network.

### 4.2 Conditional moments estimator

The count-based estimator has many limitations. In particular, it does not have a lot of granularity in choosing reactions to remove from the network; all reactions with the same count must be removed simultaneously. Thus it is possible that a reduced model given by the count-based estimator could be further reduced. This means its performance is also dependent on the specific system being studied; systems with important rare events will be difficult to meaningfully reduce with the count-based technique.

We introduce a principled method for reducing reactions from the network by finding alternative networks that contain fewer reactions while minimizing the differences between the probabilistic distributions of their resulting molecular concentration trajectories over time. In order to describe this difference between stochastic models, we consider how molecular concentration changes between consecutive timesteps for each model. The change in concentration of all molecular species follows a distribution corresponding to a linear combination of Poisson random variables – the sum over all reactions of the number of times each reaction happens times each reaction's effect on molecular concentrations. This distribution will have a mean and a covariance, both conditional on the molecular concentration at the current timestep. We seek to minimize the differences between the reduced model *versus* the full model on the conditional means and covariances of the change in concentration, at all relevant starting concentrations. This is formulated as a loss function between the two evaluated at sampled timesteps/starting molecular concentrations, which we describe in detail below.

Note that in contrast to the previous step in our framework where we were building the full stochastic model from molecular dynamics data and both reactions and rates were parameters, here when determining the reduced stochastic model we consider the set of reactions to be fixed, and only the rates are parameters.

We eliminate reactions by setting their corresponding reaction rate to 0. We would thus like to find a set of reaction rates such that as many rates as possible are 0 (*e.g.* the set of reaction rates is ***sparse***), while minimizing the loss function between the reduced model and the full stochastic model.

We sample relevant regions of concentration space from the full stochastic model by running Gillespie simulations and recording molecular concentrations at fixed time intervals Δ*t*. In this work, we use one Gillespie simulation to produce the sample data. In practice, we can improve the estimator by sampling from multiple Gillespie simulations, or also including samples from the projected molecular dynamics trajectory.

#### 4.2.1 Notation

Consider a chemical reaction network with *m* molecules and *r* reactions, which we have sampled *T* + 1 times with a timestep of Δ*t* between samples. At each sampled step *t*, denote by **X**(*t*) ∈ ℝ^*m*^ the vector of molecular concentrations. Also, denote by *y*_*t*+1_ = **X**(*t*+1) – **X**(*t*) the vector of changes in molecular concentration between timesteps.

Let the matrix *R* ∈ ℝ^*m*^ × ℝ^*r*^ denote the matrix of elementary reactions, where each reaction is represented by the column vector *R*_*j*_. For example, consider the following two reactions involving molecular species A, B, C:A + B → C2C → A

Then the corresponding *R*_*j*_ are
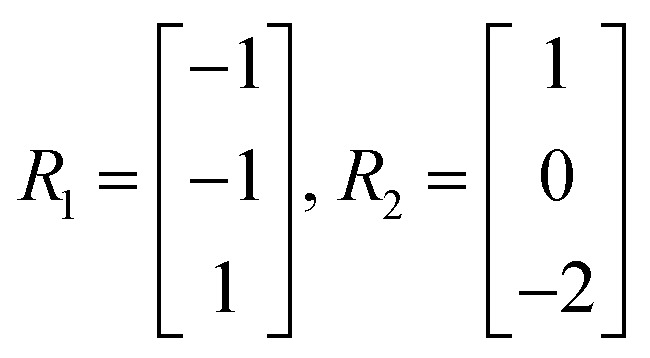
where the entries of each *R*_*j*_ correspond to the stoichiometric coefficients of molecules A, B, and C, respectively in each reaction.

#### 4.2.2 Linear system

First, we note that at any given timestep, *y*_*t*+1_ can be expressed as the sum of all reactions that occurred between timesteps *t* and *t* + 1. In vector notation, this is8
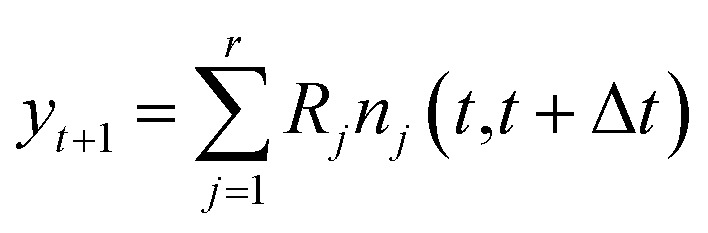

9
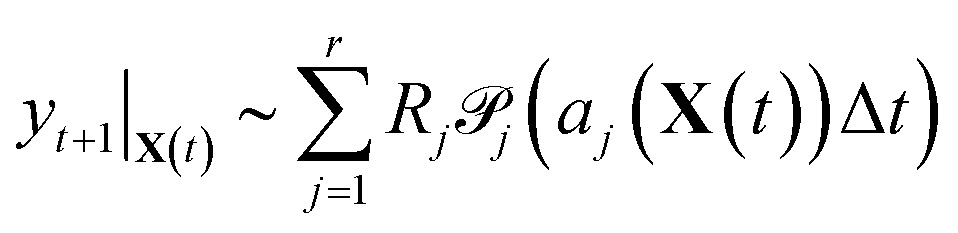



It is in general very hard to solve for the distribution of this random variable exactly since it is a linear combination of Poisson random variables. However, it is not difficult to compute the first and second moments. By linearity of expectation, we have that10
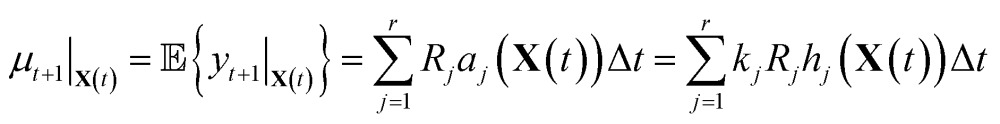



Similarly, for the covariance we note that all of the 𝒫_*j*_ are independent of each other, so that

11 *Σ*_*t*+1_|_**X**(*t*)_ = *RΛ*(**X**(*t*))*R*^*T*^

where *Λ*(**X**(*t*)) is a diagonal matrix whose *j* ^th^ entry is *a*_*j*_(**X**(*t*))Δ*t* = *k*_*j*_ *h*_*j*_(**X**(*t*))Δ*t*.

Now that we have linear expressions for the means and covariances of *y*_*t*+1_|_**X**(*t*)_ in terms of **k**, we can set up an optimization problem to find the sparsest **K**_sp_ such that the distributions of *y*_*t*+1_|_**X**(*t*)_ are minimally affected in the least squares sense over means and covariances. Given *T* + 1 samples from a simulation, we have *T* samples of *y*_*t*+1_|_**X**(*t*)_, which allows us to construct *T* pairs of *μ*_*t*+1_|_**X**(*t*)_, *Σ*_*t*+1_|_**X**(*t*)_ which we stack into a large vector to obtain12
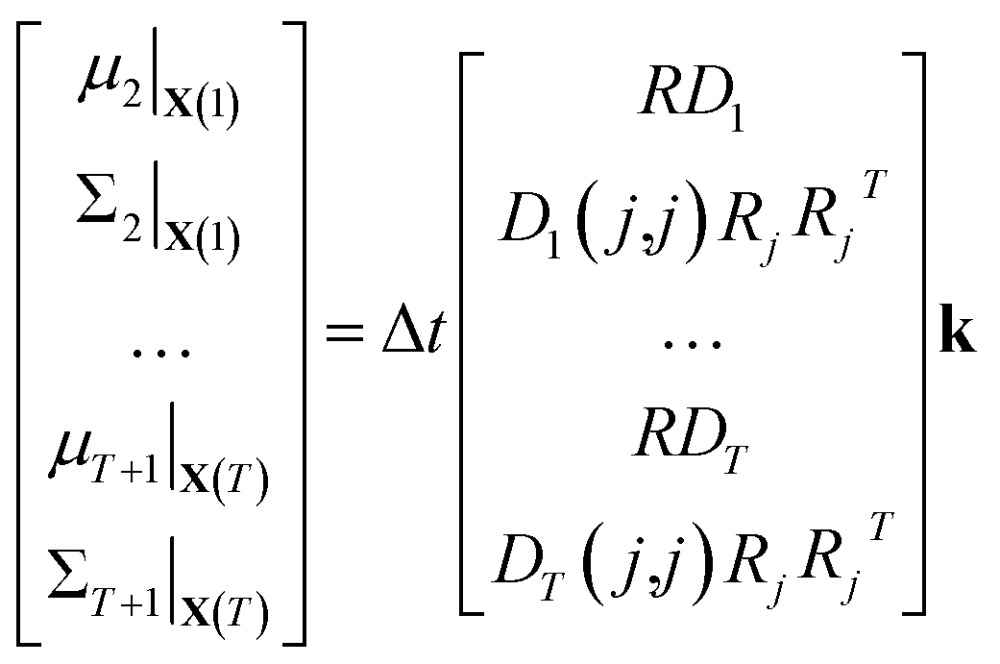
where *D_t_* is a diagonal matrix whose *j* ^th^ entry is *h_j_*(**X**(*t*)) and the expression *D_t_*(*j*,*j*)*R_j_R_j_* ^T^ indicates that each column corresponds to the expansion of that expression into a vector for a particular *j*. We then scale the variable **k** by the maximum likelihood estimated **k**_est_ from the full stochastic model (see Section 3.2.2). This is both to increase the numerical stability of the problem, and to ensure that model reduction treats each reaction equally regardless of scaling. We then have that13
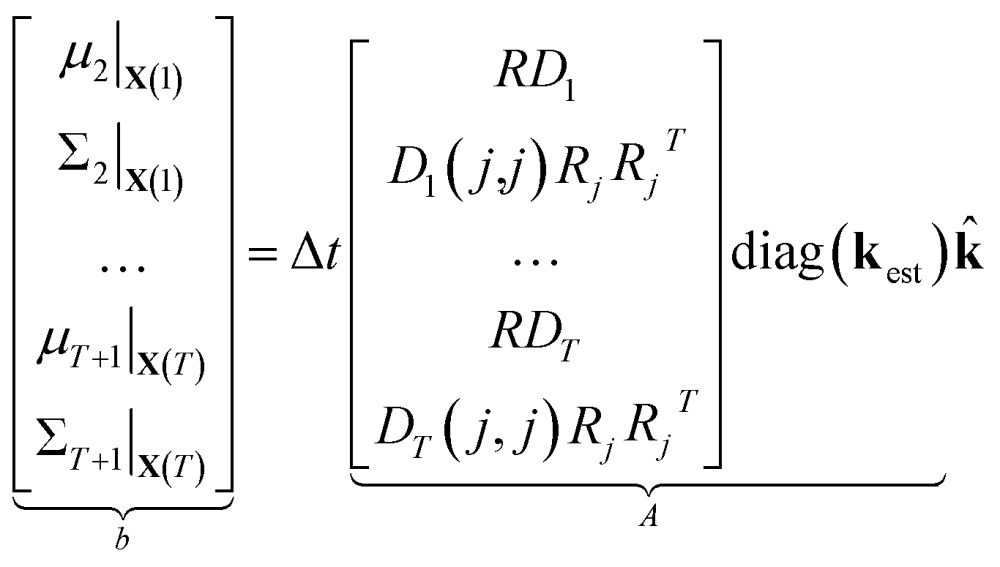

14 ⇒*b* = *A***k**

We compute *μ*_*t*_ and *Σ*_*t*_ using **k**_est_ so that, by construction, *b* = *A* **1** and **k** = **1** is an exact solution of this system. *A* is generally a very tall matrix. The number of rows of *A* is *T* × (*m* + *m*(*m* + 1)/2). That is, for each sampled timestep (1, …, *T*), there are *m* rows for *μ*_*t*_ and *m*(*m* + 1)/2 rows for the upper triangular entries of symmetric *Σ*_*t*_ (recall *m* is the number of distinct molecular species). The number of columns of *A* is the number of reactions *r*.

The rank of *A* is difficult to determine *a priori* since it depends on the particular values and zero patterns of each *D*_*t*_, but we note that it is usually undetermined until there are a large number of datapoints. This is because *R* is underdetermined since every column must be stoichiometric. In general, the larger the number of timesteps *T*, the greater the rank of *A*; it is possible for *A* to be full rank for large enough *T*.

#### 4.2.3 Loss function

In the previous step we have expressed the mean and covariance of the change in molecular concentrations *y*_*t*_ as a linear function of the dimensionless scaled reaction rate coefficients **k**_*j*_. Now, for a given complexity *λ* we attempt to find the set of **k**_*j*_ that minimizes the least squares error in the means and covariances over all sampled timesteps, subject to the constraint that a maximum of *λ* of the rate coefficients **k**_*j*_ are nonzero.

#### 4.2.4 IQP formulation

Our estimator for the set of sparse reaction rate coefficients can be expressed as the solution to an integer programming problem15
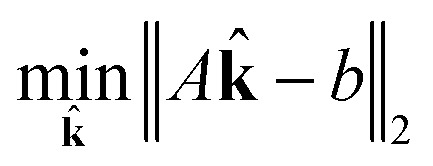

16 subject to **k** ∈ {0, 1}

17
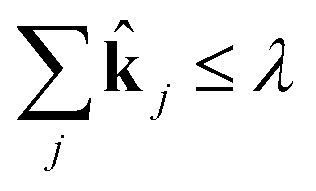



Integer programs are NP hard, and computationally expensive in practice (possibly even intractable) for very large reaction networks. However, we note that since we have a simple quadratic objective and only linear constraints in addition to the integer constraint, there exist modern algorithms that can solve the problem in very reasonable time in practice. We use TOMLAB and the CPLEX branch and cut algorithm in this work.^31^

#### 4.2.5 LASSO formulation

For very large systems, however, the IQP formulation may still be prohibitively expensive. Convex relaxation of integer programs has emerged in recent years as an important way to compute good approximate solutions to these difficult problems in guaranteed polynomial time. The convex relaxation for the IQP problem above is given by18
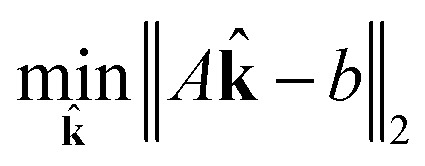

19 subject to **k** ∈ [0, 1]

20
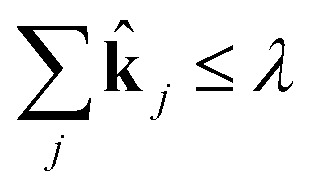



After solving for **k**, we apply a threshold to eliminate numerically zero rates. If **k**_*j*_ < *ε*, then we set **k**_*j*_ = 0. In this work we choose *ε* = 0.01. This large value of *ε* is possible because we observe a phase separation in the **k**_*j*_; they are either close to 1 or approximately 0, and *ε* is chosen to reflect the observed boundary. The reaction rate coefficients for reaction *j* are then set to be **k**_est_·st**k**_*j*_. Thus we remove reactions if we find that the estimated reaction rates can be reduced by more than 99%.

This is a convex problem because the objective is quadratic and the constraints are all linear. Since the **k**_*j*_ are allowed to be any real value between 0 and 1, the solution is no longer guaranteed to be *λ*-sparse. However, we note that it is closely related to the LASSO problem and L_1_ regularization,^32,33^ which has been the subject of intensive study in recent years due to its ability to promote sparsity. The constrained form of the LASSO is given by21
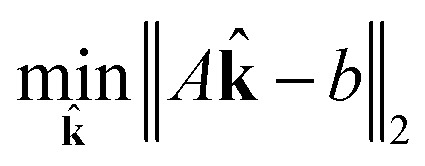

22
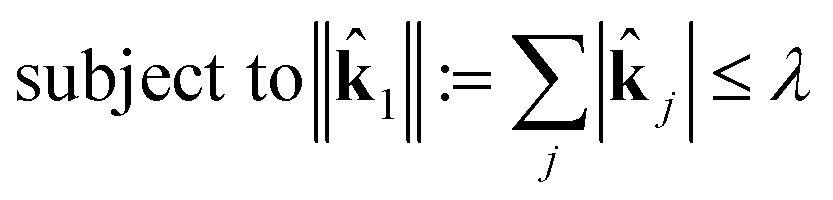



**Fig. 9:**
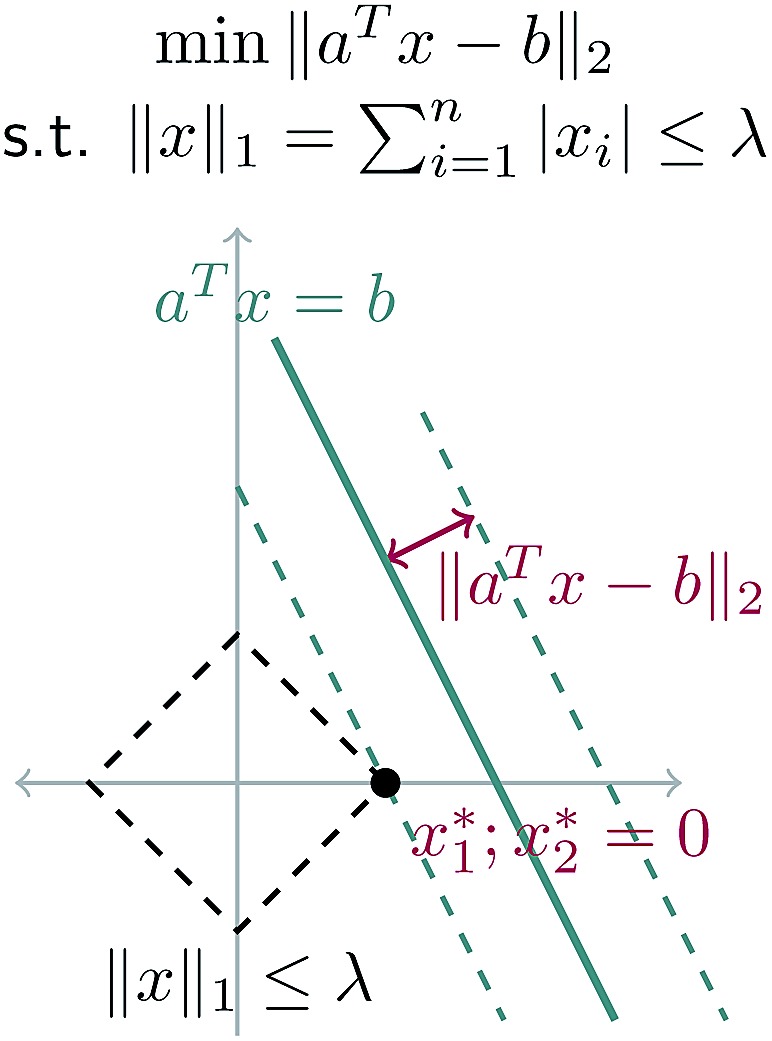
Schematic of L_1_ regularization. We try to find the best approximate solution to *a* ^*T*^ *x* = *b* that satisfies the L_1_ constraint. With high probability, this leads to a solution on a vertex of the L_1_ ball, which has sparsity (zero entries) in the solution.

A geometric interpretation for why L_1_ regularization tends to lead to sparse solutions is depicted in Fig. 9. The shape of the L_1_ constraint means that with high probability, a solution to the least squares problem will satisfy the constraint at a vertex on some coordinate axis (or hyperplane in higher dimensions), which naturally leads to zero entries in the solution.

Our problem is equivalent to the LASSO problem with an additional box constraint that **k**_*j*_ ∈ [0, 1]. Although the geometric interpretation must be modified in this case, we can see from our results that for our problem, a sparse solution is still obtained.

We note that in this formulation, the rate coefficients of the final reduced model are allowed to decrease in magnitude from their estimated values **k**_est_. This is consistent with our earlier intuition that the estimated rate coefficients that are more likely to be inaccurate, because they correspond to rarely possible reactions, are likely to be overestimates but not underestimates.

#### 4.2.6 Improving the model

We note that the estimator based on conditional moments described here is a framework upon which several variations can be built. We can adjust the number of sampled timesteps and number of sampled Gillespie simulations in order to increase the accuracy of the estimator. Computationally, we are not limited by the number of samples used to construct *A*, because our algorithm only requires knowledge of *A* ^*T*^ *A*, which can be built efficiently and corrected for stability using the singular value decomposition.

However, it might be desirable, for example, to weight the rows of *A* so that the error metric is not biased by the relative concentrations of the molecules. When row weighting is desirable, the construction of *A* ^*T*^ *W* ^*T*^ *WA* is computationally expensive, although it is still memory efficient.

#### 4.2.7 Error metric

There are several types of errors that arise from the model reduction. The first error comes from the difference between the reduced stochastic model and the full stochastic model. The second error comes from the difference between the full stochastic model and the molecular dynamics simulation of the potential energy surface. Finally, there will always be some level of error caused by stochastic fluctuations. However, we note that for any given reduced stochastic model, there may be a cancellation of errors between all three of these effects. Thus we report on the error between the reduced stochastic model and the molecular dynamics simulation. We also measure the error between the full and reduced stochastic models. When comparing stochastic models, we prefer the model with lower mean error as defined by23
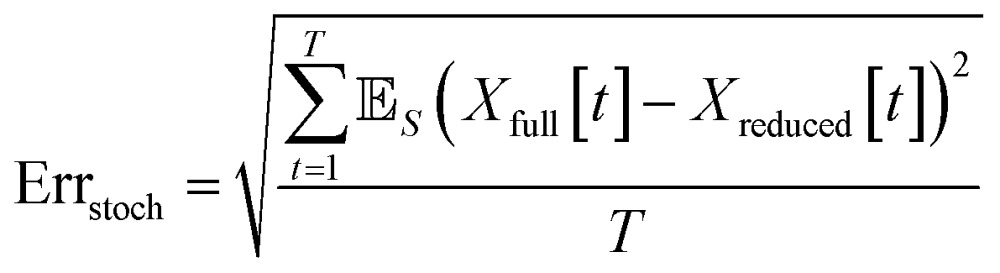
where the mean is taken over *S* stochastic simulations. When comparing molecular dynamics and the reduced stochastic models, we use the same error metric as in expression 6 above.

### 4.3 Results and discussion

**Fig. 10:**
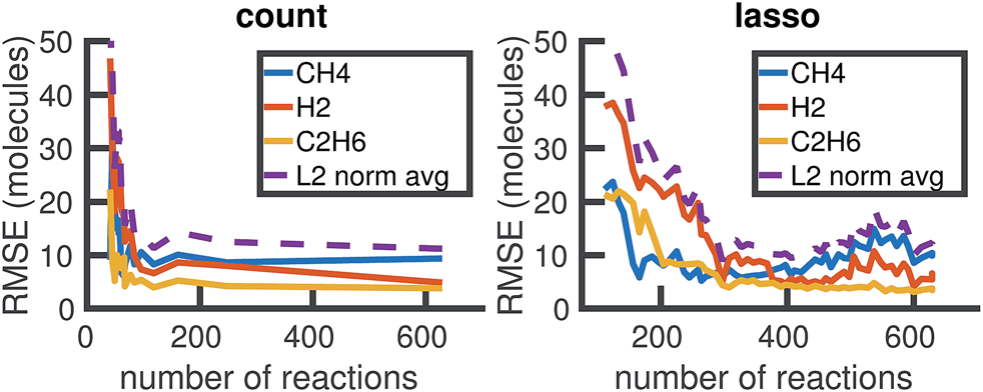
Training on the first 200 ps of molecular dynamics data, we use both the count-based and LASSO methods to reduce the chemical reaction network and compare how predictive the reduced models are when extrapolated to 500 ps of simulation time. Errors were computed using eqn (6) over *S* = 10 Gillespie simulations. The full KMC model observed in 200 ps of molecular dynamics data contains 629 reactions. The minimum overall error for CH_4_, H_2_, and C_2_H_6_ is achieved by a reduced model consisting of about 100 reactions using the count-based method and between 300 and 450 reactions using the LASSO method. Comparing with Fig. 11, however, we see that the 450 reaction network obtained by LASSO is able to capture carbon cluster growth, whereas the 100 reaction network obtained by the count-based method does not result in any carbon clusters.

**Fig. 11:**
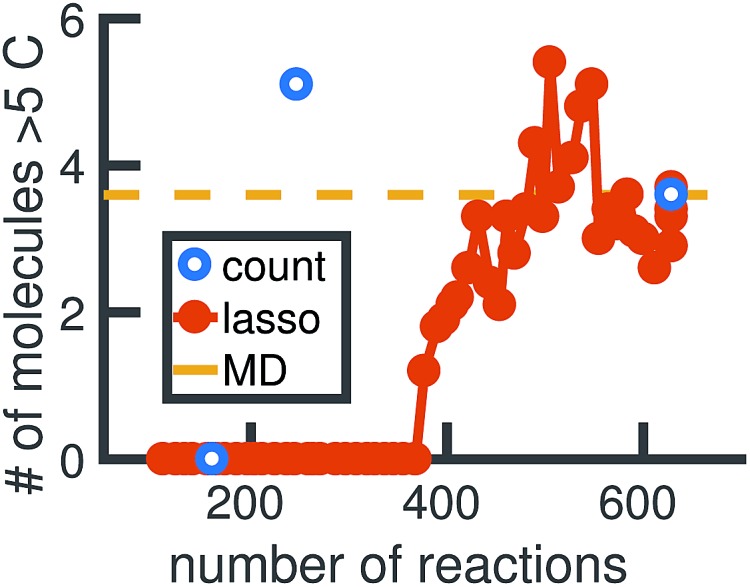
We train reduced KMC models with the first 200 ps of molecular dynamics data, and show the final number of molecules containing more than 5 carbons after 500 ps of simulation. The blue points correspond to reduced models built using the count-based method, averaged over 10 Gillespie simulations. The red line corresponds to reduced models built using the LASSO method, similarly averaged over 10 simulations. The dotted yellow line corresponds to the full KMC model averaged over 10 simulations, which is a good approximation for the MD data. Due to the lack of granularity in the count-based method, only the full model of 629 reactions is able to reasonably simulate carbon cluster growth. The next largest reduced model contains only 247 reactions, and overshoots the carbon cluster concentration. Using the LASSO method, we see that about 450 reactions are needed to reasonably model carbon cluster growth. We note that this was also among the best models for the largest concentration molecules in Fig. 10.

We begin by using the first 200 picoseconds of one molecular dynamics simulation to build a full KMC model, and comparing the predictive power of corresponding reduced models when extrapolated to 500 picoseconds of simulation time. The full model consists of 629 reactions. In Fig. 10, we see that the count-based method is able to find a reduced model with about 100 reactions that reasonably predicts the molecular concentration trajectories of CH_4_, H_2_, and C_2_H_6_, while the LASSO method requires between 300 and 450 reactions to do so. However, in Fig. 11, we see that the 100 reaction network found by the count-based method is unable to simulate the growth of any carbon clusters, which we have defined to include all molecules containing more than 5 carbon atoms. In fact, the count-based method is unable to find any reduced models that reasonably model carbon cluster growth; the only reduced model that finds any carbon cluster growth at all overshoots after 500 picoseconds. By contrast, the 450 reaction network found by the LASSO method is able to do reasonably well.

These results highlight two key differences between the simple count-based method and our data-driven LASSO method. First, the count-based method is able to find smaller reaction networks than the LASSO method that have predictive power for the largest concentration molecules, but the LASSO method is able to find reduced reaction networks that have predictive power for both the largest concentration molecules and more rare molecules such as carbon clusters. Second, the count-based method suffers from a lack of granularity. After the full model of 629 reactions, the second largest model found by the count-based method consists of only 247 reactions. This precludes the count-based method from finding any models with between 247 and 629 reactions that may capture carbon growth well, such as those found by the LASSO method. By contrast, the LASSO (and IQP) method is able to sweep parameter space with *λ* to find reduced models of any size.

We now consider model reduction using all of the more than 550 picoseconds of the available molecular dynamics data. We use one molecular dynamics simulation as input to our model reduction framework, and test the comparison between the reduced stochastic model and molecular dynamics on a different molecular dynamics simulation. This allows us to determine how well the reduced stochastic model generalizes to slightly different initial conditions and sampling of the potential energy surface. Since we have 6 MD simulations of the same system, we have 30 training-test pairs of simulations. We start comparisons after 10 ps of the molecular dynamics to allow vibrational modes to equilibrate.

**Fig. 12:**
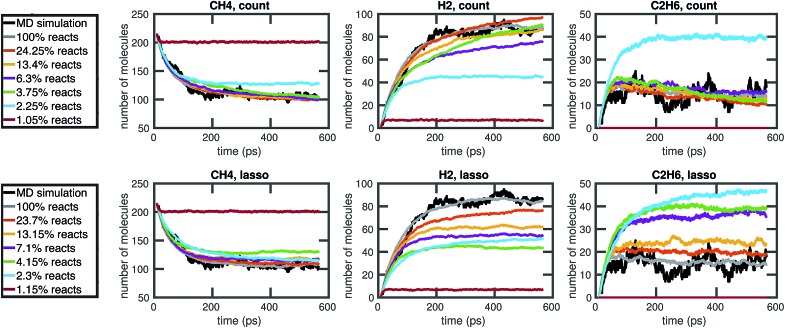
Comparison of the molecular concentration trajectories of CH_4_, H_2_, and C_2_H_6_ observed from a molecular dynamics simulation and a set of corresponding reduced stochastic models derived using the count-based method (top row) and the LASSO method (bottom row). Note that the reduced stochastic models are plotted using the mean concentration trajectories over 50 Gillespie simulations (and thus appear to have less fluctuations than the single MD simulation). The full stochastic model (100% reacts) contains 2000 reactions.

**Fig. 13:**
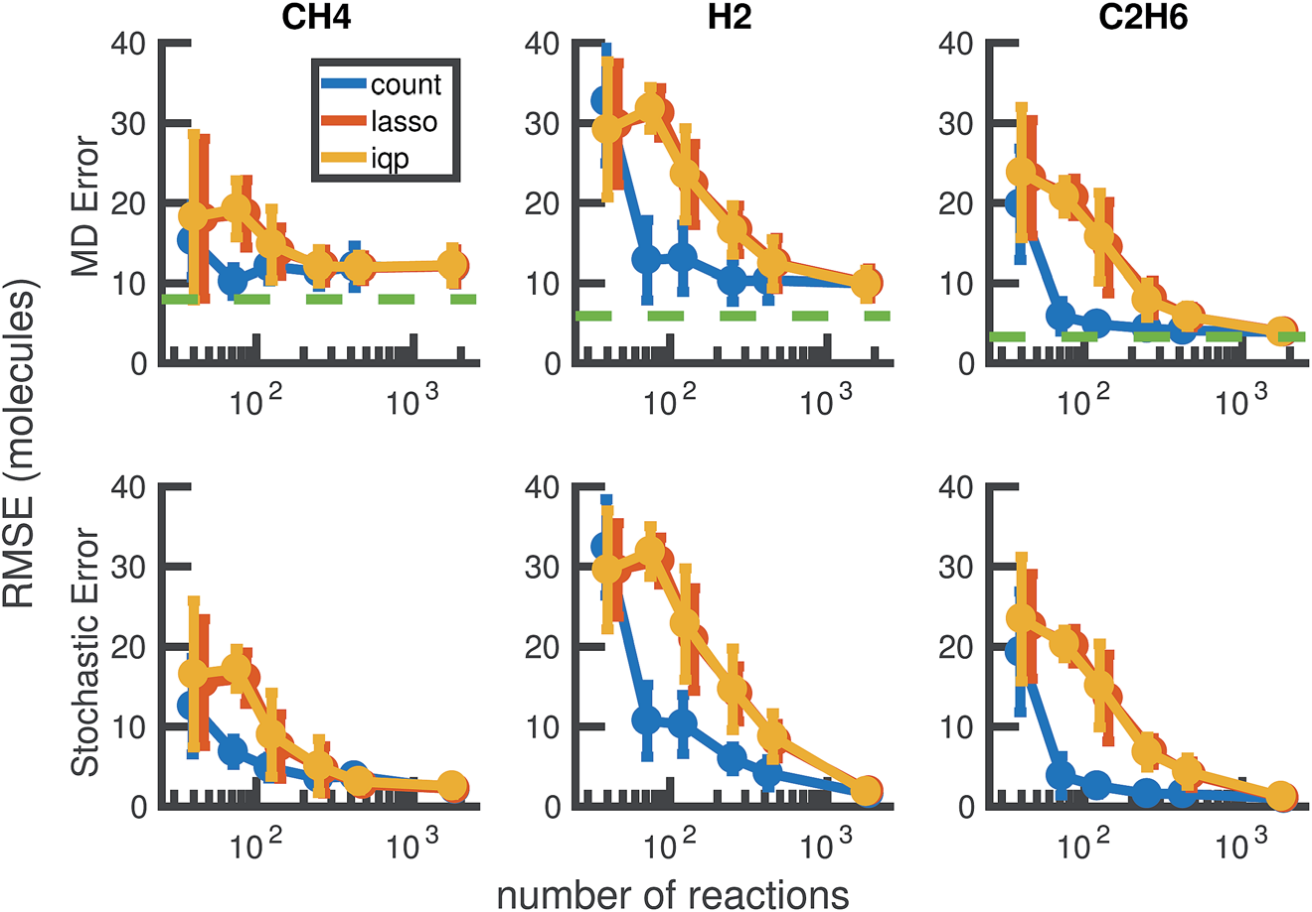
We compare the results of three model reduction methods: removing infrequent reactions (freq), solving the integer programming problem (iqp), and solving the constrained lasso problem (lasso). For each method, we adjust a single parameter *λ* over a range of values to obtain a series of increasingly small sets of reactions and corresponding reaction rates. We then simulate each reduced order model using Gillespie stochastic simulation. We compare the mean molecular concentration trajectories of CH_4_, H_2_, and C_2_H_6_ obtained over 30 Gillespie simulations with reference trajectories. In the top row, the reference trajectories are single molecular dynamics simulations. In the bottom row, the reference trajectories are the mean molecular concentration trajectories obtained from 30 Gillespie simulations over the full stochastic model. This process is repeated for all 30 pairs of training and test molecular dynamics simulations. All results above are averaged over these 30 pairs, and the error bars represent standard deviations. To provide a frame of reference for the amount of unavoidable error due to fluctuations in any single molecular dynamics simulation, the green dotted line in the top row represents the average deviation from the mean molecular dynamics trajectory among the 6 MD datasets (see Fig. 2). We can see that only around 100 reactions are needed to achieve approximately the same amount of error in CH_4_ compared to MD as the full model of almost 2000 reactions.

Our results in Fig. 12 and 13 show that the naive method of removing infrequent reactions seems to be the most predictive among reduced models of the same size for our methane system, requiring around 100 reactions out of approximately 2000 observed reactions to simulate the concentration trajectories of CH_4_ and C_2_H_6_ up to the same accuracy compared to molecular dynamics as the full stochastic model. This is a 20× reduction in the size of the model. Out of the three stable molecules considered, CH_4_, H_2_, and C_2_H_6_, the concentration of H_2_ is most sensitive to reduction in the stochastic model.

**Fig. 14:**
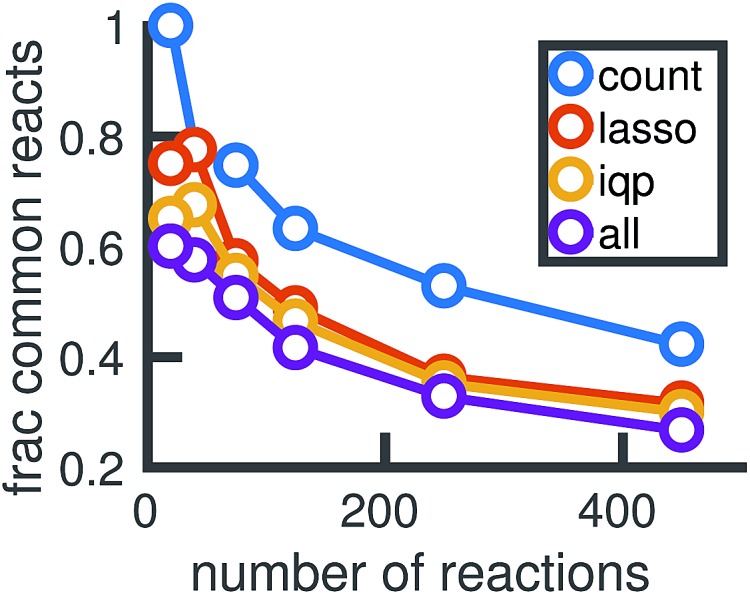
The reactions chosen by different reduced models may differ depending on the full stochastic model they were derived from, which are each constructed from a particular molecular dynamics simulation. For reduced models of different sizes, the plot shows the fraction of reactions that are common to all 6 models derived using each model reduction method from the 6 different full stochastic models corresponding to independent MD simulations. The purple line finds the percentage of reactions common to all models across all sets of data.

Fig. 14 shows how the reactions chosen for the reduced stochastic models differ across molecular dynamics simulations. For reduced stochastic models with approximately 125 reactions, only about 40% of reactions are the same across all of the different reduced models derived from different molecular dynamics simulations using our three different reduction methods, and only about 65% of reactions are the same across reduced models derived from the count-based reduction method. This suggests that there are substantial fluctuations between molecular dynamics simulations that result in differences between the reduced models. However, the similarity between reduced models increases as the sparsity of the network is increased. This suggests that there is a consistent set of important reactions.

**Fig. 15:**
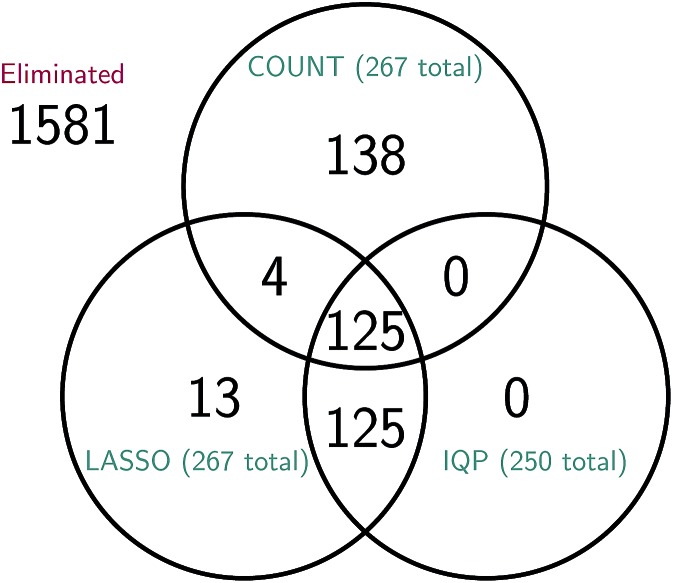
Comparison of the reactions selected by each of the three model reduction methods for a molecular dynamics simulation with 1848 total observed reactions. The total number of reactions in the reduced model given by the count-based method (COUNT) is 267; the reduced models given by the integer program (IQP) and its convex relaxation (LASSO) have 250 and 267 reactions, respectively. While it is difficult to compare reduced models due to differences in the number of reactions selected, we have attempted to choose similarly sized models here; the IQP and LASSO models were derived using the same *λ* and subsequently the most similarly sized RARE model was chosen. We can see that roughly half of the 250 to 267 reactions in each model are chosen by all three methods. As expected, the IQP and LASSO models are much more similar to each other, with the 250 reactions chosen by IQP a proper subset of the 267 reactions chosen by LASSO.

It is difficult to directly compare the set of reactions selected by the three reduction methods, since the reduced models are generally of different sizes for the same amount of error. However, we can attempt to make a comparison between reduced models of similar sizes. In Fig. 15 we consider a molecular dynamics simulation with 1848 total observed reactions, and three similarly sized reduced stochastic models corresponding to each of our three reduction methods. For *λ* = 250, the integer program gives a reduced model with exactly 250 reactions, the constrained LASSO results in 267 reactions, and the count-based model most similar in size contained 267 reactions. We can see that the integer program and constrained LASSO are more similar to each other than they are to the naive method, but only about half of the reactions in each reduced model are chosen by all three methods. This suggests that in some sense, there are opportunities to identify even better models, and in particular we may be able to gain additional sparsity by choosing some intersection of their selected reactions.

While it is easy to understand how the count-based method chooses the order in which reactions are eliminated, the optimization problems posed by the integer program and constrained LASSO are a bit less straightforward. We can see from Fig. 14 that the yellow and red lines, corresponding to IQP and LASSO respectively, match quite closely to the purple line representing the set of reactions chosen by all three methods. This means that most of the reactions chosen by all of the LASSO and IQP models are also chosen by the count-based model; that is, the reactions that LASSO and IQP consistently choose to keep in the reaction network tend to be among the most frequent reactions. However, they also seem to be removing some frequent reactions in favor of less frequent reactions that have a larger effect on the least squares error in the mean and variance of the changes in molecular concentration. Since least squares errors are sensitive to large elements and we compute the least squares error over all timesteps, these infrequent reactions only need to make a significant contribution at some timesteps, rather than at most timesteps, in order to be noticed and retained by IQP and LASSO.

In the IQP problem (expression 15), the least squares loss function used in our conditional moments estimator includes all of the molecules. This is by design in order to better capture nonlinear effects in the system. However, it may not result in the sparsest network possible for the selected important molecules. The constrained LASSO problem also suffers from this effect. In contrast, the count-based method is by construction likely to model the highest concentration molecules the best. This is because the most frequent reactions tend to involve the highest concentration molecules (recall that the propensity function *a*_*j*_(**X**(*t*)) is proportional to the concentration of the reactants). If the selected important molecules are also the highest concentration molecules, it is likely to do better than IQP and constrained LASSO.

In Table 1 we explore the ability of these model reduction methods to reveal important reaction pathways for methane decomposition. For a single molecular dynamics simulation, we find the smallest reduced model with a root mean square error approximately less than or equal to the minimum model error between the full stochastic model and molecular dynamics (around 10 molecules). We find that the count-based method can be used to construct a reduced model that does this with only 63 reactions. This reduced model exhibits an average test error of 8 molecules when simulating the remaining 5 molecular dynamics simulations it was not trained on. All of the selected reactions are observed within the first 150 ps of the molecular dynamics simulation.

**Table 1:** Important reactions for methane CH_4_. Reactions are numbered by the order in which they appear in the molecular dynamics simulation. The list of 63 reactions is computed from the count-based method for model reduction trained over a single molecular dynamics simulation. All of the selected reactions are observed within the first 150 ps of the molecular dynamics simulation. This reduced network exhibits an average error of 8 molecules when tested on the other 5 molecular dynamics simulations

No.	Reaction
2	C1 H3 3(H–C) + H1 ⇒ C1 H4 4(H–C)
4	C1 H4 4(H–C) ⇒ C1 H3 3(H–C) + H1
5	C1 H4 4(H–C) + H1 ⇒ C1 H5 4(H–C) 1(H–H)
6	C1 H5 4(H–C) 1(H–H) ⇒ C1 H3 3(H–C) + H2 1(H–H)
8	C1 H3 3(H–C) + C1 H4 4(H–C) ⇒ C2 H7 8(H–C)
9	C2 H7 8(H–C) ⇒ C1 H3 3(H–C) + C1 H4 4(H–C)
11	C2 H6 1(C–C) 6(H–C) + H1 ⇒ C2 H7 1(C–C) 7(H–C)
12	C2 H7 1(C–C) 7(H–C) ⇒ C2 H6 1(C–C) 6(H–C) + H1
13	C2 H6 1(C–C) 6(H–C) ⇒ C2 H5 1(C–C) 5(H–C) + H1
14	H1 + H1 ⇒ H2 1(H–H)
15	C1 H4 4(H–C) + C2 H5 1(C–C) 5(H–C) ⇒ C1 H3 3(H–C) + C2 H6 1(C–C) 6(H–C)
17	C1 H3 3(H–C) + C1 H4 4(H–C) ⇒ C2 H7 1(C–C) 7(H–C)
18	C2 H7 1(C–C) 7(H–C) ⇒ C1 H3 3(H–C) + C1 H4 4(H–C)
21	C1 H4 4(H–C) + H1 ⇒ C1 H3 3(H–C) + H2 1(H–H)
22	C1 H3 3(H–C) + C1 H3 3(H–C) ⇒ C2 H6 1(C–C) 6(H–C)
27	C1 H3 3(H–C) + H2 1(H–H) ⇒ C1 H4 4(H–C) + H1
28	C1 H3 3(H–C) + H2 1(H–H) ⇒ C1 H5 4(H–C) 1(H–H)
29	C1 H5 4(H–C) 1(H–H) ⇒ C1 H4 4(H–C) + H1
35	C1 H3 3(H–C) + C2 H6 1(C–C) 6(H–C) ⇒ C3 H9 1(C–C) 10(H–C)
36	C3 H9 1(C–C) 10(H–C) ⇒ C1 H4 4(H–C) + C2 H5 1(C–C) 5(H–C)
37	C1 H3 3(H–C) + C2 H6 1(C–C) 6(H–C) ⇒ C3 H9 2(C–C) 9(H–C)
38	C3 H9 2(C–C) 9(H–C) ⇒ C1 H3 3(H–C) + C2 H6 1(C–C) 6(H–C)
42	C1 H3 3(H–C) + C2 H6 1(C–C) 6(H–C) ⇒ C1 H4 4(H–C) + C2 H5 1(C–C) 5(H–C)
43	C2 H5 1(C–C) 5(H–C) + H1 ⇒ C2 H6 1(C–C) 6(H–C)
45	C1 H4 4(H–C) + H1 ⇒ C1 H5 5(H–C)
46	C1 H5 5(H–C) ⇒ C1 H4 4(H–C) + H1
47	C1 H4 4(H–C) + C2 H5 1(C–C) 5(H–C) ⇒ C3 H9 1(C–C) 10(H–C)
48	C3 H9 1(C–C) 10(H–C) ⇒ C1 H3 3(H–C) + C2 H6 1(C–C) 6(H–C)
49	C2 H5 1(C–C) 5(H–C) ⇒ C2 H5 1(C–C) 6(H–C)
50	C2 H5 1(C–C) 6(H–C) ⇒ C2 H5 1(C–C) 5(H–C)
51	C2 H6 1(C–C) 6(H–C) + H1 ⇒ C2 H7 1(C–C) 6(H–C) 1(H–H)
52	C2 H7 1(C–C) 6(H–C) 1(H–H) ⇒ C2 H5 1(C–C) 5(H–C) + H2 1(H–H)
53	C2 H5 1(C–C) 5(H–C) ⇒ C2 H4 1(C–C) 4(H–C) + H1
58	H2 1(H–H) ⇒ H1 + H1
60	C2 H4 1(C–C) 4(H–C) + H1 ⇒ C2 H5 1(C–C) 5(H–C)
61	C2 H6 1(C–C) 6(H–C) ⇒ C1 H3 3(H–C) + C1 H3 3(H–C)
62	C3 H9 2(C–C) 9(H–C) ⇒ C3 H8 2(C–C) 8(H–C) + H1
64	C2 H6 1(C–C) 6(H–C) + H1 ⇒ C2 H5 1(C–C) 5(H–C) + H2 1(H–H)
65	C2 H5 1(C–C) 5(H–C) + H2 1(H–H) ⇒ C2 H7 1(C–C) 6(H–C) 1(H–H)
67	C2 H7 1(C–C) 6(H–C) 1(H–H) ⇒ C2 H6 1(C–C) 6(H–C) + H1
69	C1 H3 3(H–C) + C2 H5 1(C–C) 5(H–C) ⇒ C3 H8 2(C–C) 8(H–C)
71	C1 H3 3(H–C) + C3 H8 2(C–C) 8(H–C) ⇒ C4 H11 2(C–C) 12(H–C)
72	C4 H11 2(C–C) 12(H–C) ⇒ C1 H4 4(H–C) + C3 H7 2(C–C) 7(H–C)
73	C4 H11 2(C–C) 12(H–C) ⇒ C1 H3 3(H–C) + C3 H8 2(C–C) 8(H–C)
75	C1 H4 4(H–C) + C3 H7 2(C–C) 7(H–C) ⇒ C1 H3 3(H–C) + C3 H8 2(C–C) 8(H–C)
86	C2 H5 1(C–C) 5(H–C) + C2 H6 1(C–C) 6(H–C) ⇒ C4 H11 2(C–C) 12(H–C)
87	C4 H11 2(C–C) 12(H–C) ⇒ C2 H5 1(C–C) 5(H–C) + C2 H6 1(C–C) 6(H–C)
88	C3 H8 2(C–C) 8(H–C) ⇒ C3 H7 2(C–C) 7(H–C) + H1
89	C3 H7 2(C–C) 7(H–C) ⇒ C3 H7 2(C–C) 8(H–C)
90	C3 H7 2(C–C) 8(H–C) ⇒ C3 H7 2(C–C) 7(H–C)
92	C1 H3 3(H–C) + C3 H8 2(C–C) 8(H–C) ⇒ C1 H4 4(H–C) + C3 H7 2(C–C) 7(H–C)
96	C1 H4 4(H–C) + C3 H7 2(C–C) 7(H–C) ⇒ C4 H11 2(C–C) 12(H–C)
118	C1 H3 3(H–C) + C4 H10 3(C–C) 10(H–C) ⇒ C5 H13 3(C–C) 14(H–C)
119	C5 H13 3(C–C) 14(H–C) ⇒ C1 H4 4(H–C) + C4 H9 3(C–C) 9(H–C)
122	C4 H9 3(C–C) 9(H–C) ⇒ C4 H9 3(C–C) 10(H–C)
123	C4 H9 3(C–C) 10(H–C) ⇒ C4 H9 3(C–C) 9(H–C)
126	C1 H4 4(H–C) + C4 H9 3(C–C) 9(H–C) ⇒ C1 H3 3(H–C) + C4 H10 3(C–C) 10(H–C)
172	C5 H11 4(C–C) 11(H–C) ⇒ C5 H11 4(C–C) 12(H–C)
173	C5 H11 4(C–C) 12(H–C) ⇒ C5 H11 4(C–C) 11(H–C)
178	C2 H5 1(C–C) 5(H–C) + H2 1(H–H) ⇒ C2 H6 1(C–C) 6(H–C) + H1
254	C4 H10 3(C–C) 10(H–C) ⇒ C4 H9 3(C–C) 9(H–C) + H1
255	C1 H4 4(H–C) + C4 H9 3(C–C) 9(H–C) ⇒ C5 H13 3(C–C) 14(H–C)
256	C5 H13 3(C–C) 14(H–C) ⇒ C1 H3 3(H–C) + C4 H10 3(C–C) 10(H–C)

## 5 Run time of algorithms

While the molecular dynamics simulations required about four weeks of computation on a parallelized version of LAMMPS, the Gillespie stochastic simulations require only a few minutes on MATLAB.

Further model reduction of the stochastic system reduces the computation time of each Gillespie stochastic simulation step in a linear fashion, but does not automatically reduce the number of steps necessary to simulate a finite time interval. Since the number of steps is the largest contribution to the computation time of Gillespie stochastic simulation, and it is controlled by the rate of the fastest reactions, the more fast reactions that we can eliminate, the faster it will run.

We note that while solving for the reduced stochastic system with the naive method takes essentially no time, LASSO and IQP scale differently. In general IQP is NP hard, but for this problem size it is tractable, albeit slower than LASSO. The constrained LASSO takes approximately 69 seconds using Matlab's quadprog interior point solver, and its computation time is approximately constant with *λ*; its computational complexity depends only on the size of the full reaction network to be reduced, in our case approximately 2000 reactions. For a reaction network of the same size, the IQP using TOMLAB's CPLEX solver takes as much as 250 seconds; note that for large enough and small enough *λ*, the runtime decreases significantly for IQP because the feasible set is smaller and may even be faster than LASSO. However, as the total number of reactions in the full stochastic system increases, the computation time of IQP grows rapidly.

## 6 Conclusions

In this study, we find that rare events are unlikely to play an important role in the decomposition of high temperature high pressure liquid methane. Our reduced stochastic models show that only a small subset of the most frequent reactions observed from the first 150 ps of the molecular dynamics simulation are necessary to predict methane concentration over time.

The statistical framework we develop in this work to build reduced KMC models from molecular dynamics is highly automated and requires minimal system-specific parameterization. One of the key insights is that bond duration plays a crucial role in determining model complexity, and thus should be chosen to optimize the predictive power of the KMC model rather than be a fixed parameter. We show that the resulting models are able to extrapolate in time to new regions of molecular concentration space, which suggests that KMC models learned from molecular dynamics data can be used to meaningfully model chemistry that they were not trained on.

Our data-driven model reduction algorithm, perhaps not surprisingly, results in somewhat larger reaction networks than simple count-based reduction when maximizing predictive power for the largest concentration molecules. However, it is much more granular in being able to find reduced models of different sizes, and better able to capture the growth of more rare molecules. Computationally, its integer program formulation is already more efficient than existing model reduction methods based on integer programming, because it has a quadratic objective and only simple bound constraints other than the integer constraint. Replacing the integer constraint with L1-regularization further transforms the problem to be computationally tractable at large scales.

As advances in computing power and algorithms enable increasingly accurate, large-scale molecular dynamics simulations, one of the grand challenges of computational chemistry and materials science will be interpreting the large amount of generated data, and using it to build better mesoscale models. Our results from this work suggest the possibility for a genomic approach to studying complex chemistry in the future, where data from expensive molecular dynamics simulations are reused to build an increasingly large and statistically accurate database of elementary reactions and rates, from which reduced KMC models can then be quickly and systematically built for target chemical systems, obviating the need for any MD simulations.
